# ﻿*Ecdyonurusaurasius* sp. nov. (Insecta, Ephemeroptera, Heptageniidae, Ecdyonurinae), a new micro-endemic mayfly species from Aurès Mountains (north-eastern Algeria)

**DOI:** 10.3897/zookeys.1121.89613

**Published:** 2022-09-12

**Authors:** Besma M. Dambri, Nadhira Benhadji, Laurent Vuataz, Michel Sartori

**Affiliations:** 1 Department of Ecology and Environment, Faculty of Natural and Life Sciences, University of Batna 2, 05078 Fesdis, Batna, Algeria University of Batna 2 Batna Algeria; 2 Department of Hydrobiology, Institute of Biology, University of Szczecin, Felczaka street 3 c, 71- 412 Szczecin, Poland University of Szczecin Szczecin Poland; 3 Musée cantonal de zoologie, Palais de Rumine, Place de la Riponne 6, 1014 Lausanne, Switzerland Musée cantonal de zoologie Lausanne Switzerland; 4 Department of Ecology and Evolution, Biophore, University of Lausanne, 1015 Lausanne, Switzerland University of Lausanne Lausanne Switzerland

**Keywords:** Belezma National Park, COI, mayflies, new species, North Africa, taxonomy

## Abstract

*Ecdyonurusaurasius***sp. nov.**, a micro-endemic species reported from several streams within the Aurès Mountains (north-eastern Algeria), is described and illustrated at nymphal, subimaginal and imaginal stages of both sexes. Critical morphological diagnostic characters distinguishing the new species are presented, together with molecular affinities as well as notes on the biology and distribution of the species.

## ﻿Introduction

The genus *Ecdyonurus* Eaton, 1868 belongs to the Ecdyonurinae Ulmer, 1920, a subfamily with rather challenging and controversial taxonomy as genera delineation and phylogeny are still partially unsolved or in process (Kluge 2004; Wang and McCafferty 2004; Bauernfeind and Soldán 2012; Yanai et al. 2017). The identification key to genera proposed by Webb and McCafferty (2008) displayed 14 genera in the world; among them, four genera *Ecdyonurus*, *Electrogena* Zurwerra & Tomka, 1985, *Afronurus* Lestage, 1924 and *Paracinygmula* Bajkova, 1975 (sub. nom. *Nixe* Flowers, 1980; see Sartori 2014 for discussion) possess Palearctic species. The first three are the most diversified with 61, 45 and 64 species respectively worldwide (Barber-James et al. 2013; Yanai et al. 2017). Recently, the new genus *Anapos* Yanai & Sartori, 2017 was created to accommodate two Mediterranean species.

In Africa, only three Ecdyonurinae genera are present: *Ecdyonurus* is restricted to North Africa, whereas *Afronurus* and *Notonurus* Crass, 1947 are found in the Afrotropical region (Webb and McCafferty 2008; Vuataz et al. 2013).

Bauernfeind and Soldán (2012) proposed to split the West Palearctic species of the genus *Ecdyonurus* into two subgenera: *Ecdyonurus* (25 species) and *Helvetoraeticus* Bauernfeind & Soldán, 2012 (15 species), according to the arrangement of setae on the superlingua, the number of bristles on the ventral side of the labrum and the number of comb-shaped bristles on the maxilla in nymphs, as well as the shape of the apical sclerite of the male genitalia.

Currently, four taxa of this genus are reported from North Africa (Thomas 1998). Two of them are well-known species with a clear status: *Ecdyonurusrothschildi* Navás, 1929 and *Ecdyonurusifranensis* Vitte & Thomas, 1988, whereas one remains doubtful: Ecdyonurusvenosusvar.constantinicus Lestage, 1925, and the presence of *Ecdyonurusvenosus* (Fabricius, 1793) mentioned by Gauthier (1928) is still unconfirmed. All of them belong to the subgenus Ecdyonurus.

Navás (1929) described *Ecdyonurusrothschildi* from an oasis in Biskra Province, north-eastern Algeria, based on a male imago. The species was redescribed by Thomas and Dakki (1979) which gave a detailed account of the adult morphology and related it to the *E.aurantiacus* (Burmeister, 1839) species group. Later, Soldán and Gagneur (1985), proposed the first description of the nymph and an identification key to separate *E.rothschildi*, *E.dispar* (Curtis, 1834) and *E.aurantiacus* nymphs. The species is now known from all Maghreb countries and is one of the most widespread species (Boumaiza and Thomas 1995; Zrelli et al. 2016; Bouhala et al. 2020). Vitte and Thomas (1988) described *Ecdyonurusifranensis* at nymphal and adult stages from the Middle Atlas; the species has later been found in other areas of Morocco (El Alami et al. 2022).

The present study aims to examine *Ecdyonurus* populations from the Aurès region (Algeria). We collected and reared fresh material at all stages. After critical observations and comparison with other *Ecdyonurus* species, we have clearly distinguished a new Algerian endemic species.

## ﻿Materials and methods

**Figure 1.**
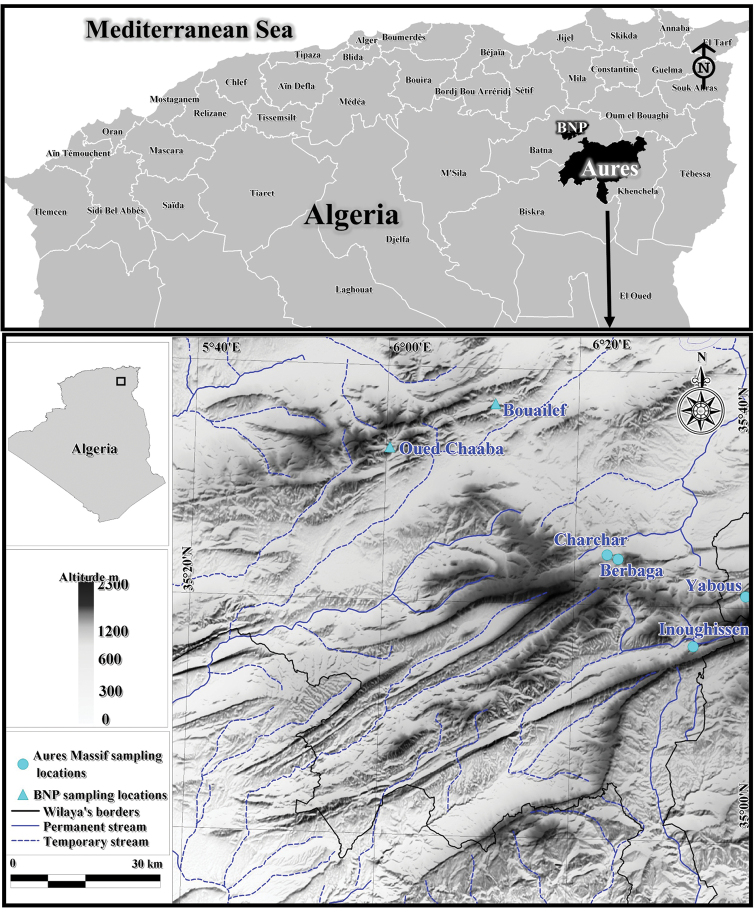
Map of the sampling sites.

The material was collected by the first author between February 2020 and November 2021 from six localities from the Aurès region; the sampling sites are located in the Belezma National Park (BNP) and the Western Aurès Massif (Fig. 1). The region is characterized by a semi-arid climate with cold winters and very hot and dry summers. Sampling was performed using a standard benthic net using the kick-sampling method. Imagos and subimagos were obtained by rearing mature nymphs from the Charchar, Yabous and Berbaga sites. All specimens were preserved in 96% ethanol in the field and stored in the laboratory at 4 °C.

The physical and chemical parameters of the water was measured in situ for each sampling site using a multi-probe. The following variables were measured: average water depth, bed width, current velocity with a FLOWATCH flowmeter; conductivity, water temperature and pH using an Adwa AD32 tester and a HANNA HI1271 pH electrode; while dissolved oxygen was recorded using a Lutron PDO-519 Dissolved Oxygen Meter.

### ﻿Morphological analysis

Morphological characteristics for the description of the new species were used according to Hrivniak et al. (2018). Pictures of habitus were made using a Canon EOS 6D camera and the Visionary Digital Passport imaging system (formerly available and distributed by Dun Inc., Virginia), and processed with Adobe Photoshop Lightroom ver. 4.4. and Helicon Focus ver. 5.3. Four nymphs were dissected in Cellosolve (2-Ethoxyethanol) with subsequent embedding in Euparal medium and mounting on slides. Microscopic pictures were taken using an Olympus BX51 microscope coupled with an Olympus SC50 camera; pictures were enhanced with the stacking software Olympus Stream Basic ver. 2.3.2. and Adobe Photoshop ver. 21.2.2.

### ﻿Molecular analysis

Five specimens belonging to the new species as well as five specimens of *Ecdyonurusrothschildi* were used for DNA extraction to get a 658 bp fragment of the mitochondrial cytochrome oxidase I gene (COI) (see Table 1). DNA extraction, PCR amplification, sequencing and alignment construction were performed according to Benhadji et al. (2020) or Martynov et al. (2022). One sequence of *E.rothschildi* was retrieved from GenBank, as well as two sequences of *E.aurantiacus* and two of *E.dispar*. Three *Electrogena* sequences were chosen as the outgroup. We estimated the evolutionary divergence within and between our new species and the other *Ecdyonurus* species using the COI genetic distances. Both pairwise distance between all sequences and mean distance between and within species were calculated in MegaX (Kumar et al. 2018; Stecher et al. 2020) under the Kimura 2-parameter (K80) substitution model (Kimura 1980). We then applied the recently developed species delimitation method ASAP (Assemble Species by Automatic Partitioning; Puillandre et al. 2021) to our COI data set using the graphical web-interface available at https://bioinfo.mnhn.fr/abi/public/asap/asapweb.html. This distance-based method is similar to the popular ABGD (Automatic Barcode Gap Discovery; Puillandre et al. 2012) approach but has the advantage of providing a score (i.e. asap-score) that indicates the most likely species delimitation. Pairwise genetic distances were computed under the K80 model, and all other settings were set to default. Because ASAP outputs produced two partitions with equal asap-scores, we favored the partition with the smallest p-value.

**Table 1.** Sequenced specimens of *E.aurasius* sp. nov. and *Ecdyonurusrothschildi* with collection data and nomenclature of sequences used in the molecular study.

Species	Specimen catalogue number	Stage	Locality	GPS coordinates	Date	GenBank ID	GenSeq Nomenclature
*Ecdyonurusaurasius* sp. nov.	GBIFCH 01119302	Male imago	Algeria, Wilaya de Batna, Berbaga	35°24'01N, 6°24'31"E	5.xi.2021	ON920531	genseq-2 COI
*Ecdyonurusaurasius* sp. nov.	GBIFCH 01119304	Male imago	Algeria, Wilaya de Batna, Charchar	35°24'22"N, 6°23'21"E	17.x.2021	ON920532	genseq-2 COI
*Ecdyonurusaurasius* sp. nov.	GBIFCH 00673191	Nymph	Algeria, Wilaya de Batna, Charchar	35°24'22"N, 6°23'21"E	23.vi.2019	ON920533	genseq-2 COI
*Ecdyonurusaurasius* sp. nov.	GBIFCH 00673192	Nymph	Algeria, Wilaya de Batna, Charchar	35°24'22"N, 6°23'21"E	23.vi.2019	ON920534	genseq-2 COI
*Ecdyonurusaurasius* sp. nov.	GBIFCH 00673193	Male imago	Algeria, Wilaya de Batna, Charchar	35°24'22"N, 6°23'21"E	23.vi.2019	ON920535	genseq-2 COI
*Ecdyonurusrothschildi*	GBIFCH 00763579	Nymph	Algeria, oued Cherf, Dbabcha	36°13'00"N, 7°19'05"E	18.x.2019	ON920536	genseq-4 COI
*Ecdyonurusrothschildi*	GBIFCH 00763578	Nymph	Algeria, oued Bougous, Oum Ali	36°37'53"N, 8°18'54"E	23.i.2019	ON920537	genseq-4 COI
*Ecdyonurusrothschildi*	GBIFCH 01116263	Nymph	Morocco, Draa, Mgoune downstream	31°20.07'N, 6°10.82'W	22.x.2021	ON920538	genseq-4 COI
*Ecdyonurusrothschildi*	EC-CH0	Nymph	Algeria, Tafna, Chouly 0	34°47'20"N, 1°13'07"W	19.xii.2015	ON920529	genseq-4 COI
*Ecdyonurusrothschildi*	EC-CH1	Nymph	Algeria, Tafna, Chouly 1	34°49'15"N, 1°10'55"W	19.xii.2015	ON920530	genseq-4 COI
*Ecdyonurusrothschildi*		Nymph	Tunisia		vii.2009	HG935040	genseq-4 COI

Finally, we conducted a Bayesian inference gene tree reconstruction in MrBayes ver. 3.2.7a (Ronquist et al. 2012), using the best evolutionary model (GTR + Γ + I) selected in JModelTest ver. 2.1.10 (Darriba et al. 2012) following the second-order Akaike information criterion (AICc). We used five substitution scheme and six gamma categories, with all other parameters set to default. To accommodate different substitution rates among COI codon positions, we analyzed our data set in two partitions, one with first and second codon positions and one with third positions (1 + 2, 3). Two independent analyses of four MCMC chains run for one million generations with trees sampled every 1000 generations were implemented, and 100 000 generations were discarded as a burnin after visually verifying run stationarity and convergence in Tracer ver. 1.7.2 (Rambaut et al. 2018). The consensus tree was visualized and edited in iTOL 6 (Letunic and Bork 2021).

Material is deposited in the following institutions:

**FEEL-UB2** Functional Ecology and Environmental Laboratory, University Batna 2, Algeria;

**IB-US**Institute of Biology, University of Szczecin, Poland;

**MZL**Museum of zoology, Lausanne, Switzerland.

## ﻿Results

### ﻿Molecular analysis

**Figure 2.**
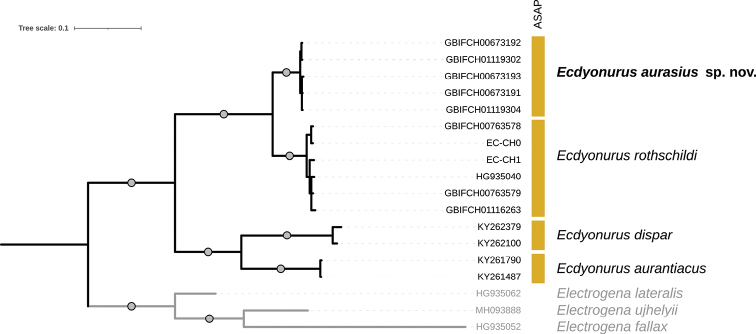
Bayesian majority-rule consensus tree reconstructed from the CO1 data set. Tips labelled with GBIF and EC-CH codes indicate newly sequenced specimens, other codes correspond to previously published GenBank sequences. Vertical boxes indicate species delimitation hypotheses according to the ASAP analysis. The outgroups are represented in grey. Circles on branches indicate Bayesian posterior probabilities > 0.95.

The COI ingroup data set was 100% complete (no missing data) and included 25% of parsimony informative sites. The COI gene tree grouped the five sequences of *Ecdyonurusaurasius* sp. nov. into a well-supported monophyletic clade, and was supported as a distinct species in the ASAP analysis (Fig. 2). The K80 mean genetic distance within the five *Ecdyonurusaurasius* sp. nov. COI sequences was 0.14%. As expected, all other included species were also recovered as distinct species with high node supports. The K80 mean genetic distance between *Ecdyonurusaurasius* sp. nov. and the other three species of *Ecdyonurus* ranged from 7.6% (mean distance to *E.rothschildi*) to 20.1% (mean distance to *E.aurantiacus*), with a minimum distance of 7.1% between GBIFCH01119302 / GBIFCH00673192 and EC-CH0 sequences.

### ﻿Morphological analysis

#### Heptageniidae Neddham, 1901

**Ecdyonurinae Ulmer, 1920**

##### ﻿ Ecdyonurus aurasius

Taxon classificationAnimaliaEphemeropteraHeptageniidae

Dambri, Benhadji & Sartori sp. nov.

11A25021-4118-5EC5-B4CF-3CFE86311118

https://zoobank.org/0A552D79-3329-4CCA-9724-D01492F82D7B

###### Material.

***Holotype*.** Algeria • male imago in ethanol, with its corresponding nymphal and subimaginal exuviae, Wilaya de Batna, Charchar, 35°24'22"N, 6°23'21"E, 1340 m. a.s.l., 09 Nov. 2021, B. Dambri coll. (GBIFCH01128855) [MZL] • ***Paratypes*.** 1 male imago, with its nymphal and subimaginal exuviae (GBIFCH01128846), 1 female imago, with its nymphal and subimaginal exuviae (GBIFCH01128858), [MZL]; 6 female imagos [IB-US], same data as holotype; 1 male imago, 1 male subimago [IB-US], 2 female imagos, 7 male subimagos [FEEL-UB2], 06 Nov. 2021; 1 male imago [IB-US], 1 male imago [FEEL-UB2], 20 Oct. 2021; 1 male imago, with its nymphal and subimaginal exuviae (GBIFCH01119304), 1 female imago, with its nymphal and subimaginal exuviae (GBIFCH01128861) [MZL], 17 Oct. 2021; 1 female imago with its subimaginal exuvia, 1 female subimago (GBIFCH01128849) [MZL], 15 Oct. 2021; 1 female subimago, 1 male subimago [IB-US], 1 male subimago (GBIFCH01128853) [MZL], 2 nymphs [FEEL-UB2], 10 Oct. 2021; 3 nymphs [IB-US], 2 nymphs (GBIFCH01128857), 1 nymph on slide (GBIFCH01119301) [MZL], 09 Oct. 2021; 7 nymphs [IB-US], 15 nymphs [FEEL-UB2], 18 Jun. 2020; 15 nymphs [IB-US], 18 nymphs [FEEL-UB2], 5 nymphs (GBIFCH01128850) [MZL], 3 Mar. 2020; same locality, B. Dambri coll; 10 nymphs (GBIFCH00832138), 2 nymphs on slide (GBIFCH00673191-GBIFCH00673192), 1 male imago (GBIFCH00673193), 1 male imago, 1 female imago, 2 female subimagos (GBIFCH00832125), 23 Jun. 2019, same locality, L. Kechemir coll. et leg. [MZL]

***Other paratypes*.** Algeria • Wilaya de Batna, Berbaga, 35°24'01N, 6°24'31"E, 1445 m. a.s.l., 1 male imago, with its nymphal and subimaginal exuviae (GBIFCH01119302), 1 female subimago with its nymphal exuvia (GBIFCH01128848) [MZL], 5 Nov. 2021; 1 male imago (GBIFCH01128852), 2 nymphs (GBIFCH01128847), 1 nymph on slide (GBIFCH01119303) [MZL], 13 nymphs [IB-US], 5 nymphs [FEEL-UB2], 4 Nov. 2021; 1 male imago [IB-US], 12 nymphs [FEEL-UB2], 30 Nov. 2020; 1 nymph [IB-US], 16 nymphs [FEEL-UB2], 03 May 2020; 1 male imago [IB-US], 10 nymphs [FEEL-UB2], 02 Mar. 2020, B. Dambri coll. Algeria • Wilaya de Khenchela, Yabous, 35°21'11"N, 6°38'35"E, 1420 m. a.s.l., 2 female imagos [IB-US], 2 female subimagos, 3 nymphs [FEEL-UB2], 22 Oct. 2021; 1 female subimago [IB-US], 2 nymphs [FEEL-UB2], 1 female imago with its subimaginal exuvia, 1 female subimago (GBIFCH01128854) [MZL], 13-14 Oct. 2021; 1 male imago with its subimaginal exuvia GBIFCH01128851), 1 female imago with is subimaginal exuvia (GBIFCH01128845) [MZL], 12 Oct. 2021; 5 nymphs [IB-US], 1 female imago, 2 male subimagos, 6 nymphs [FEEL-UB2], 09 Oct. 2021; 4 nymphs [IB-US], 2 nymphs [FEEL-UB2], 1 nymph (GBIFCH01128859) [MZL], 20 Jul. 2020; 2 nymphs [IB-US], 19 nymphs [FEEL-UB2], 1 nymph (GBIFCH01128856) [MZL], 02 Jun. 2020; 1 female subimago with its nymphal exuvia [IB-US], 8 nymphs [FEEL-UB2], 09 May 2020; 1 female subimago [IB-US], 15 nymphs [FEEL-UB2], 08 Mar. 2020; 1 female subimago with its nymphal exuvia [IB-US], 3 nymphs [FEEL-UB2], 23 Feb. 2020, B. Dambri coll. Algeria • Wilaya de Batna, Inoughissen, 35°16'42"N, 6°32'34"E, 1670 m. a.s.l., 1 nymph (GBIFCH01128863) [MZL], 07 Jul. 2020; 1 nymph (GBIFCH01128865) [MZL], 18 Apr. 2020, B. Dambri coll.

###### Other material.

Algeria • Wilaya de Batna, oued Chaâba, 35°33'03"N, 6°00'22"E, 1262 m. a.s.l.,1 nymph [IB-US], 17 Jun. 2020; 3 nymphs [IB-US], 10 nymphs [FEEL-UB2], 1 nymph (GBIFCH01128860) [MZL], 20 Apr. 2020, B. Dambri coll. Algeria • Wilaya de Batna, Bouailef, 35°37'01"N, 6°11'17"E, 1060 m, 1 nymph [IB-US], 08 Mar. 2020, B. Dambri coll.

###### Etymology.

Aurès mountains were coined by the Berber people as Awras, meaning tawny; translated by the Romans as *Aurasiusmons*; *aurasius* is a noun in apposition.

###### Description.

**Figure 3.**
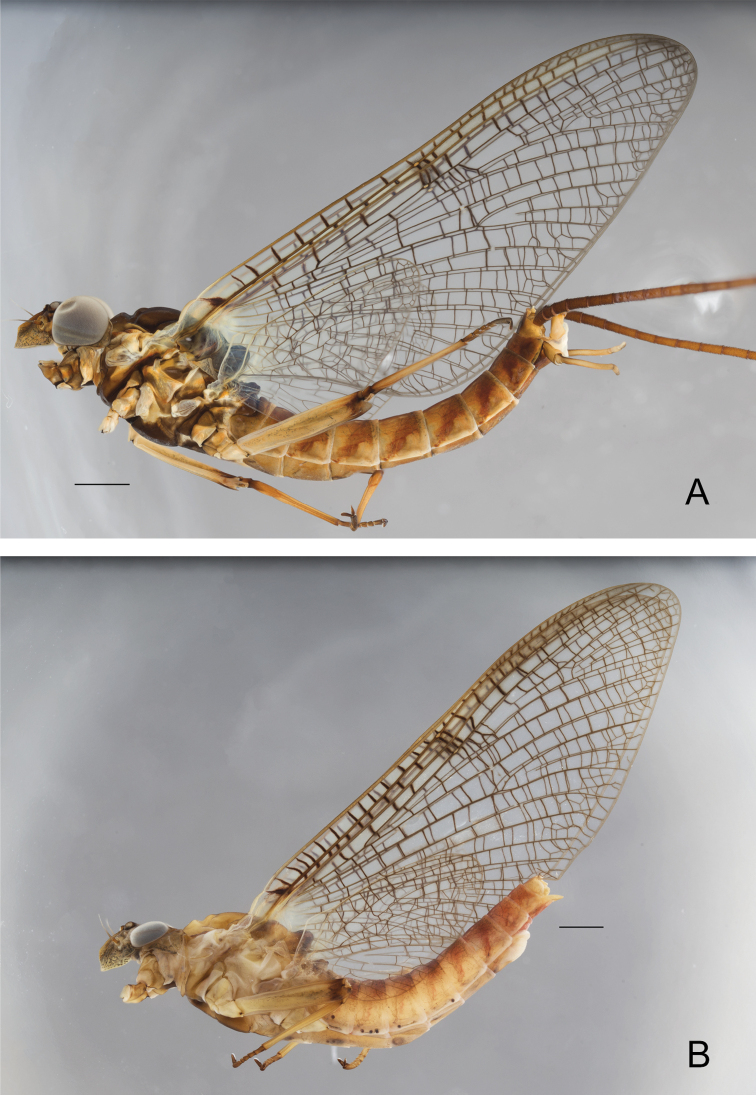
*Ecdyonurusaurasius* sp. nov., adults in lateral view **A** male **B** female. Scale bar: 1 mm.

**Male imago** Size: body length: 9.0–9.8 mm; forewing length 9.1–10.9 mm; cerci broken. General body color distinctly brown to reddish-brown (Fig. 3A).

***Head*.** Light brown, clypeal plate with blackish maculations; eyes grayish blue separated by a distance equal to the diameter of the frontal ocellus; a brownish lateral stripe present at one third of the ventral side; ocelli apically whitish-yellow, dark brown basally; antennae with scapus medium brown, flagellum grayish brown.

***Thorax*.** Pronotum medium brown; mesonotum dorsally dark brown; ventrally with basisternum and furcasternum also dark brown, laterally with spiracles and pleura yellowish-brown. ***Wings*.** Forewings hyaline, C, Sc and R_1_ longitudinal veins medium brown with transverse veins fringed with brown; first transversal vein in the costal field surrounded by a dark brown maculation; others longitudinal veins dark brown, as transversal veins; pterostigmatic area milky, with 15–20 medium brown, simple and forked cross veins. Hind wings same color as forewings. ***Legs*.** Fore legs markedly darker than middle and hind ones, brown to reddish-brown; fore femora only slightly darker than tibiae and tarsi; fore legs 8.25–9.4 mm; femur:tibia:tarsi proportion: femur 2.11–2.54 mm; tibia 2.46–2.68 mm; tarsal segments 2.68–4.18 mm; T1 = 0.66–0.73 mm; T2 = 0.99–1.08 mm; T3 = 0.87–1.06 mm; T4 = 0.67–0.77 mm; T5 = 0.49–0.54 mm; gradation of tarsal segments: 2 > 3>4 > 1>5. Middle and hind legs yellowish-brown; dorsal face of femora washed with gray; distal part of femora and proximal part of tibiae dark brown; tarsi darker than tibiae; middle legs 5.16–5.49 mm; femur:tibia:tarsi proportions: femur 2.39–2.49 mm; tibia 1.87–1.91 mm; tarsal segments 0.9–1.09 mm; hind legs 4.97–5.71 mm; femur:tibia:tarsi proportions: femur 2.53–2.82 mm; tibia 1.65–1.93 mm; tarsal segments 0.79–0.96 mm.

**Figure 4.**
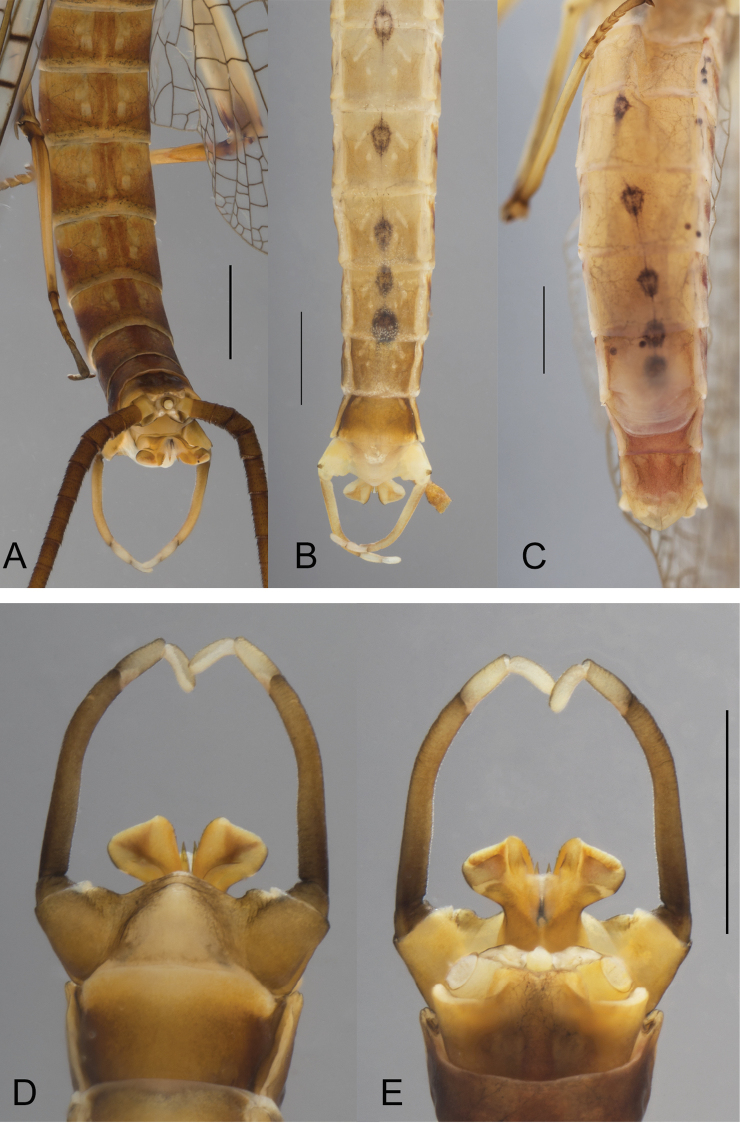
*Ecdyonurusaurasius* sp. nov., abdomen of adults **A** male in dorsal view **B** male in ventral view **C** female in ventral view **D** male genitalia in ventral view **E** male genitalia in dorsal view. Scale bar: 1 mm.

***Abdomen*.** General color brown to rusty tawny. Terga light tawny to rusty tawny. Tergum I dark brown, terga II-VII reddish-brown with two median pairs of light markings, proximal pair elongated and slightly divergent, distal pair subparallel to body axis (Fig. 4A). Segments II-VIII with rusty-brown lateral stripes stretching from anterior to posterior margin of the segment (Fig. 3A) and connected dorso-posteriorly (Fig. 4A); terga VII–X slightly darker that other ones; tergum X reddish-brown, yellowish-brown posteriorly. Abdominal sterna yellowish to light brown, with two pairs of light markings, the proximal pair elongated, and divergent, distal pair rounded (Fig. 4B). Sterna VIII-IX darker. Nervous ganglia well visible and tinted with purple on sterna II–VII. Cerci brown, with joints of segments blackish.

**Figure 5.**
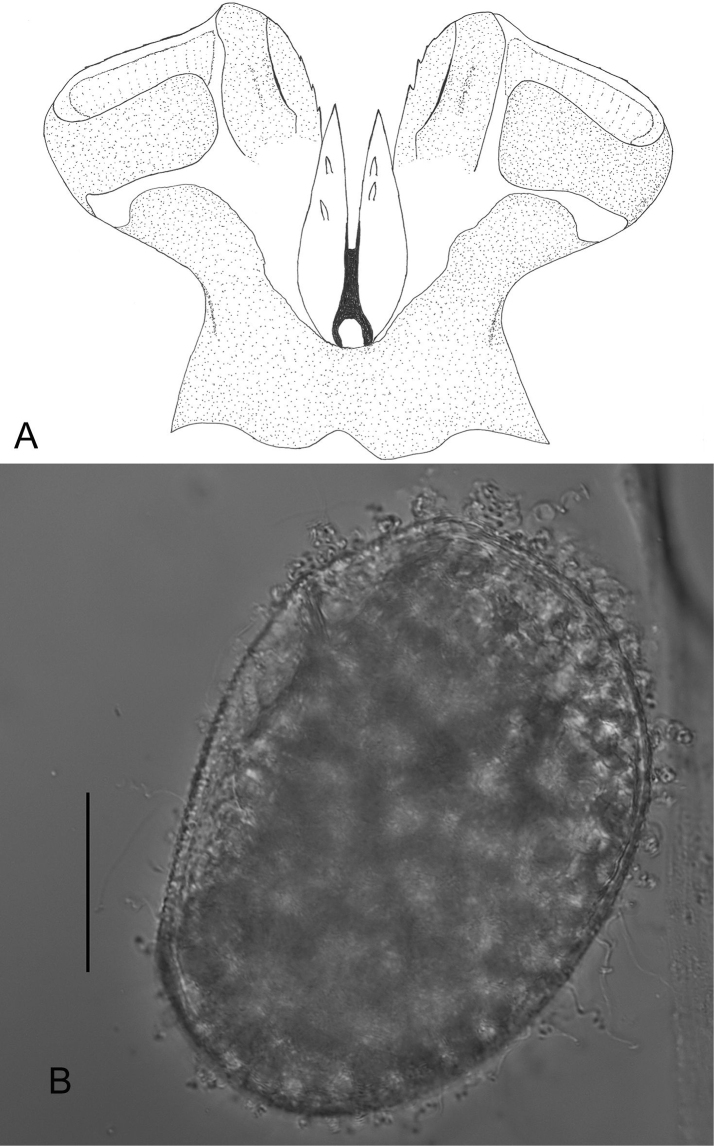
*Ecdyonurusaurasius* sp. nov. **A** penes in dorsal view **B** egg. Scale bar: 0.05 mm.

***Genitalia*.** Styliger plate medium brown, lighter in the middle, strongly convex, with two small bumps near gonostyli base; first segment of gonostyli dark brown, second and third lighter (Fig. 4D). Penis lobes yellowish-brown to brown moderately expanded laterally, outer margin rather quadratic (Fig. 4D, E). Basal and lateral sclerites brown, darker than apical sclerite (Fig. 4E). Lateral sclerite rather quadratic slightly larger on inner side; apical sclerite with few medium sized teeth on inner margin (Fig. 5A); basal sclerite outer margin smooth, without teeth. Titillators straight, yellowish-brown, darker on outer margin, with two spines on the dorsal face.

**Female imago.** Size: body length: 9.9–13.3 mm; forewings length: 10.5–12.9 mm; cerci length: 17.9–21.3 mm. General color of body similar to that in male imago, markedly paler. ***Head*.** yellowish-brown; eyes grayish. ***Thorax*.** Prothorax yellowish gray to brown. Mesothorax dorsally pale, yellow to yellowish-brown, basisternum and furcasterum medium brown. ***Abdomen*.** Terga yellowish laterally and tawny to rusty tawny dorsally. Terga I-VIII with central longitudinal rusty tawny parallel bands and lateral stripes (Fig. 3B). Abdominal sterna yellowish to light brown, especially VIII-IX, segments I–VII generally with two central light short strokes; nervous ganglia strongly tinted with purple on sterna II-VII. Subgenital plate large, whitish and angular, reaching two third of sternum VIII length; subanal plate acutely rounded (Fig. 4C). Cerci brown, with joints blackish.

**Female subimago.** Size: body length: 12.0–12.4 mm; forewings length: 12.3 mm; cerci length: 14.0–14.8 mm. Measurements and body color similar to female imago; thorax and abdomen slightly paler. Wings dull grey.

**Male subimago.** Size: body length: 9.8–10.5 mm; forewings length: 10.5–11.4 mm; cerci length: 13.3–26.9 mm. Head brown to reddish-brown. Eyes grayish blue. Ocelli as in male imago. Antennae yellowish, brown basally, same than in male imago. Fore legs darker than middle and hind ones. Fore femora intensively brown distally. Middle and hind legs uniformly yellowish gray to yellow. Wings dark gray. Abdominal terga similar to male imago. Sterna slightly lighter than terga. Protuberances of styliger plate well marked, slightly yellowish, gonostyli intensively brown, yellow to whitish-yellow apically. Typical shape of penis already well apparent. Cerci brown.

**Mature nymph.** Size: body length: up to 7.12 mm for male and 9.6 mm for female; cerci slightly longer than body length. General body color yellowish-brown with pale yellowish markings.

**Figure 6.**
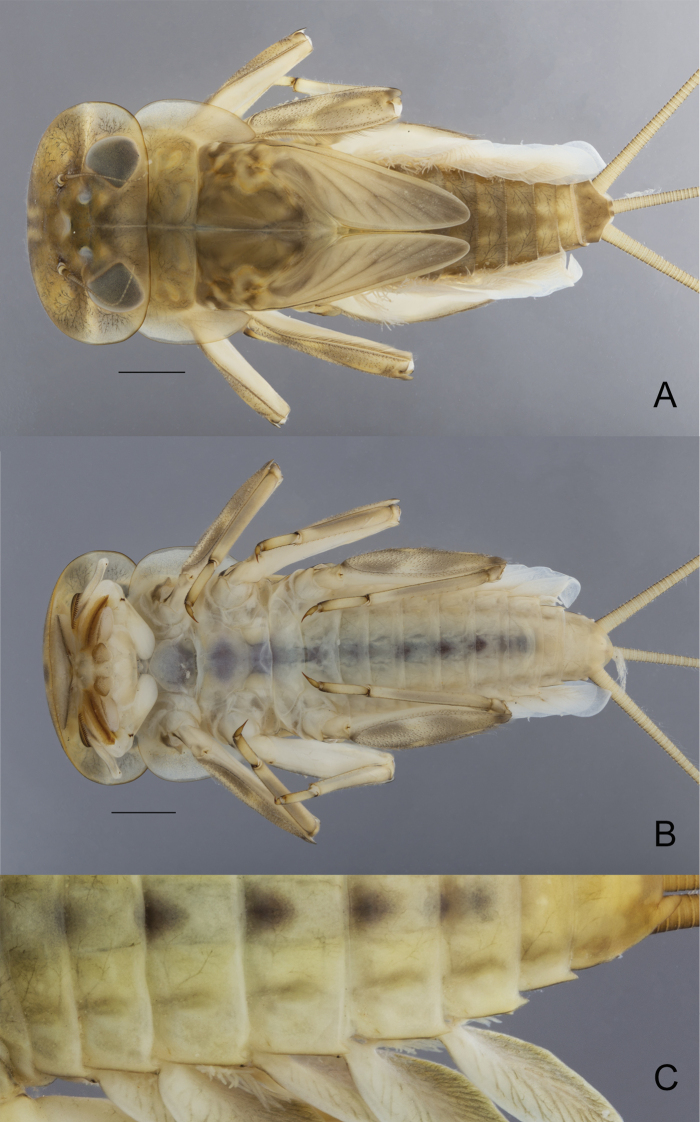
*Ecdyonurusaurasius* sp. nov., nymphal habitus **A** dorsal view **B** ventral view **C** posterolateral expansions of the abdomen. Scale bar: 1 mm.

***Head*.** Mean width/length ratio 1.4–1.6, yellowish-brown to brown, with two central light spots near fore margin, and two whitish stripes along the dehiscence line (Fig. 6A). Eyes blackish grey; ocelli whitish grey, antennae with scape and pedicel medium brown; flagellum yellowish-brown.

**Figure 7.**
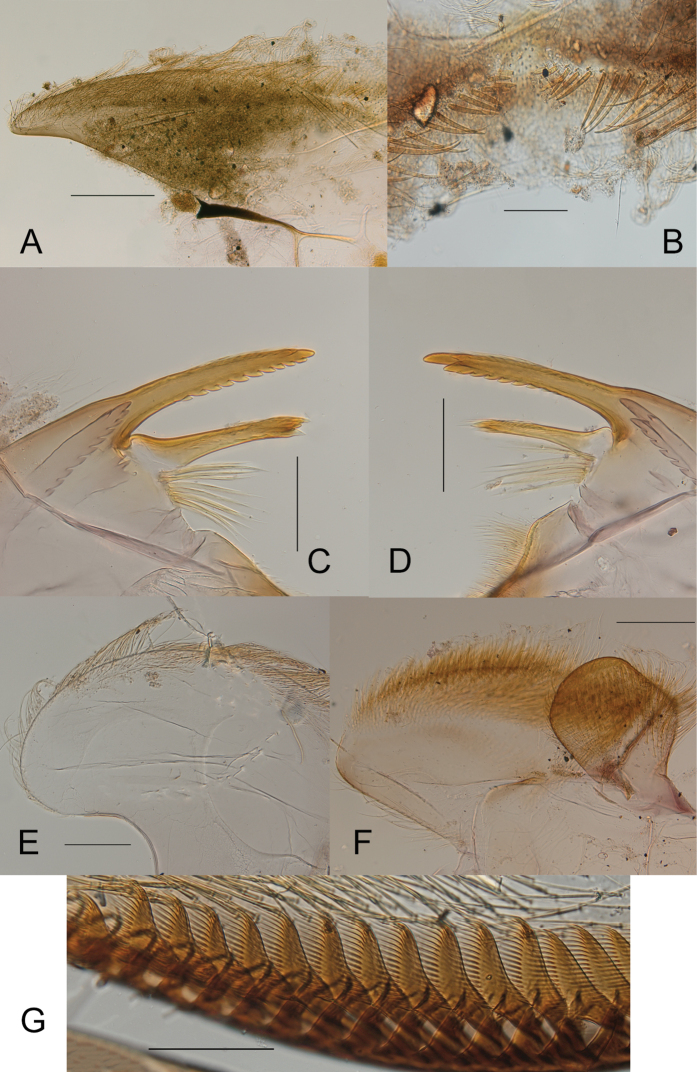
*Ecdyonurusaurasius* sp. nov., nymphal mouthparts: **A** hemi-labrum **B** detail of anteromedial part of labrum in ventral view **C** left mandible **D** right mandible **E** left half of hypopharynx **F** left half of labium **G** comb-shaped setae at the crown of the galea-lacinia. Scale bars: 0.2 mm (**A, F**); 0.1 mm (**B–E, G**).

***Mouthparts*.** Labrum. Mean length /labrum insertion length ratio 1.58; tips slightly turned backwards (Fig. 7A); anterior margin with a median single row of stout setae (Fig. 7B). Right mandible with prostheca composed of 8–11 feathered bristles; kinetodontium (inner incisor) much shorter than (outer) incisor (Fig. 7D). Left mandible with prostheca composed of 9–11 feathered setae, kinetodontium subequal in length to incisor. Hypopharynx with lingua quadratic with dorsal margin slightly concave in the middle; superlingua well developed, with long hair-like setae on outer margin becoming shorter and less dense near apex (Fig. 7E). Labium typical of the genus; glossae markedly rhomboid, with inner margin straight or slightly concave (Fig. 7F). Crown of the galea-lacinia of maxilla with 16–22 comb-shaped setae; median setae with ca 15–17 teeth (Fig. 7G). Maxillary palps 3–segmented, second segment slightly longer than third one.

***Thorax*.** Pronotum. Mean width/length ratio 4.2–5.0, yellowish-brown to brown; lateral projections ca as long as the length of the pronotum; with lateral margin regularly convex, and tip slightly pointed (Fig. 6A). Mesonotum medium brown with yellowish markings.

**Figure 8.**
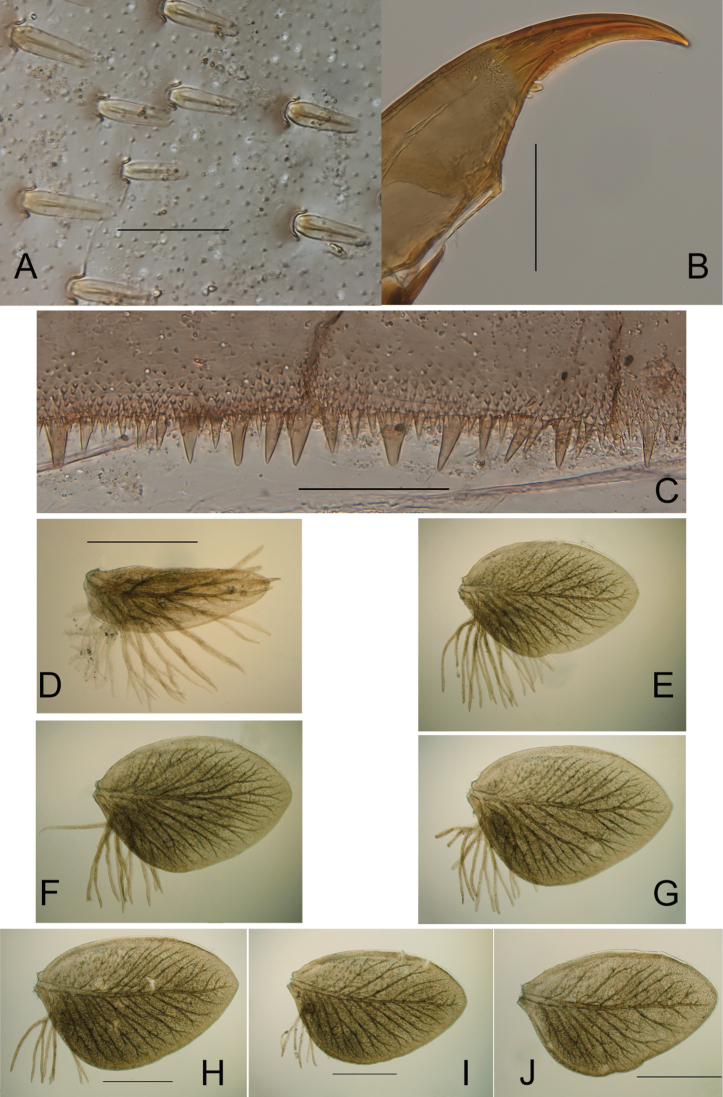
*Ecdyonurusaurasius* sp. nov., nymphal structures **A** stout setae on the dorsal surface of femora **B** claw **C** posterior margin of tergum IV **D–J** Gill I to VII. Scale bars: 0.05 mm (**A**); 0.1 mm (**B–J**); 0.5 mm (**E–G** same bar as **H**).

***Legs*.** Yellowish-brown to brown; dorsal surface of femora yellowish-brown washed with grayish brown; uniformly yellowish white ventrally. Tibiae yellowish-brown. Tarsi brownish. Middle and hind legs coloration similar to fore legs. Fore femora 2.0–2.2 times longer than wide; fore tibiae subequal in length to femora. Middle femora 2.2–2.3 times longer than wide; tibiae 0.8–0.9 times femora length. Hind femora 2.3–2.4 longer than wide; tibiae 0.80–0.85 times femora length. Mid- and hind femora length 1.1–1.3 times fore-femora length. Stout setae on dorsal surface of femora similar on all legs, elongated with subparallel margins, tip truncate or slightly rounded (Fig. 8A); claws elongated and hooked with 2–4 small denticles (Fig. 8B).

***Abdomen*.** Terga brownish gray; on terga II–VIII two centrally elongated yellowish spots increasing in size posteriorly and fused on tergum IX; tergum X uniformly medium brown (Fig. 6A). Abdominal sterna yellowish white, nervous ganglia tinted with purple. Posterior margin of terga with large pointed marginal teeth alternating with medium and short ones, and several rows of microdenticles above the margin (Fig. 8C). Posterolateral projections short, weakly sclerotized, reaching from slightly above 1/7 to 1/5 of the length of the following segment (Fig. 6C). Gills grayish brown with distinct brown and developed tracheation; gill I tongue-shaped, gills II–VII leaf-shaped, asymmetrical, gills III-IV slightly longer than wide (Fig. 8D–J). Cerci and paracercus yellowish-brown; each segment with a row of pointed stout setae.

***Egg.*** Length 165–175 µm; width 120–130 µm; numerous KCT’s densely arranged at one pole (Fig. 5B); chorionic surface covered with micro granulations.

## ﻿Discussion

*Ecdyonurusaurasius* sp. nov. belongs to the subgenus Ecdyonurus by the shape of the apical sclerite of male genitalia and the single row of stout setae on the ventral side of the labrum. However, this species presents some intermediate characters between the subgenera *Ecdyonurus* and *Helevetoraeticus*; the number of comb-shaped setae on the crown of the galea-lacinia is generally less than 20 in *Ecdyonurus* s.s., whereas our species exhibits a range from 16 to 22 setae; the setae on the lateral margin of superlingua are supposed to be long, including the tip, whereas in our species, those at the tip are shorter. We can also add the posterolateral projections on the abdomen which are very short, and the nervous ganglia tinted in purple, two characters not frequent in *Ecdyonurus* s.s. but more common in *Helvetoraeticus*. Nevertheless, we are confident that our new species belongs to the subgenus Ecdyonurus.

By the shape of the penis lobes and the posterolateral projections of the abdomen, *E.aurasius* sp. nov. is closely related to *E.aurantiacus*, *E.dispar*, *E.rothschildi*, and *E.ifranensis*. The first two are considered as Mediterranean faunal elements, expanding to Central Europe or even the British Islands for *E.dispar* (Bauernfeind and Soldán 2012). The nymph of *E.aurasius* sp. nov. can be separated from those of *E.aurantiacus* and *E.dispar* by the nervous ganglia tinted with purple, and the tongue-shaped gill I, from *E.dispar* also by the shape of the stout setae on the dorsal surface of femora (acute and pointed in the latter). The new species presents more affinities with the two other North African endemics but can be distinguished from *E.rothschildi* by the much longer pronotal projections, the shape of the stout setae on the dorsal surface of femora (pointed in the latter), the shape of the gills (more symmetrical in *E.rothschildi*) and the shape of the glossae ((inner margin rounded and convex in *E.rothschildi*). *Ecdyonurusaurasius* sp. nov. differs from *E.ifranensis* by the shape of the labrum (less broad in *E.ifranensis*), the shape of the stout setae on the dorsal surface of femora (pointed in *E.ifranensis*), and the shape of the glossae similar to *E.rothschildi*. In males, *E.aurasius* sp. nov. differs from *E.rothschildi*, *E.dispar* and *E.aurantiacus* by the compound eyes separated and not touching (character not stated in *E.ifranensis* description), from *E.aurantiacus* and *E.dispar* by the posterior margin of the basal sclerite smooth, and from *E.ifranensis* by the first transversal vein in the costal field surrounded by a dark brown maculation (the same in *E.rothschildi*), and by the shape of the posterior margin of the basal sclerite rounded (straight in *E.ifranensis*). It is also worth noting that *E.aurasius* sp. nov. differs from the two other North African species by the nervous ganglia tinted in purple in female imagos, whereas they are colorless in *E.rothschildi* and *E.ifranensis*.

### ﻿Distribution and biology

**Figure 9.**
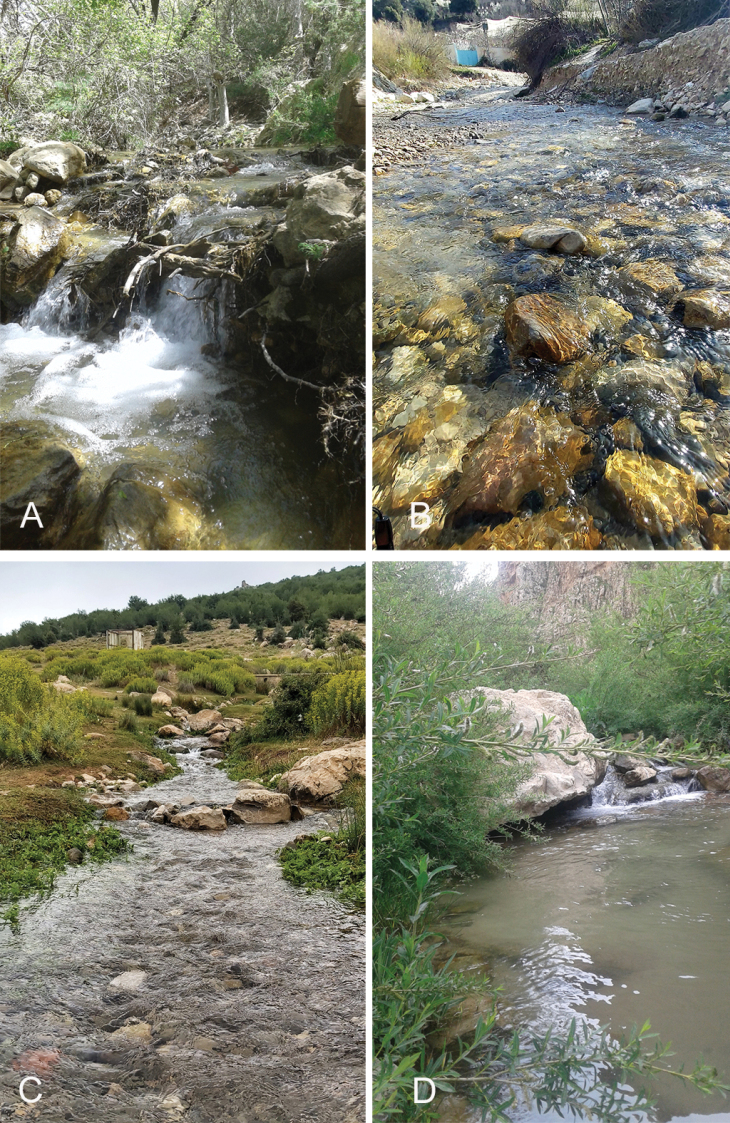
Sampling sites where *Ecdyonurusaurasius* sp. nov. was collected **A** Oued Chaâba **B** Inoughissen **C** Charchar **D** Berbaga (photos Besma Dambri).

*Ecdyonurusaurasius* sp. nov., as known so far, is restricted to the Aurès region. The species has been recorded from only six localities in the Western Aurès area; most habitats are located in the highest part of the streams, within altitudes ranging from 1010 to 1800 m a.s.l. These sites are represented by small mountain watercourses with gravel substrate (Fig. 9). The average annual water temperature ranges from 5 °С to 18 °С with high concentration of dissolved oxygen (6.5 to 9.35 mg/L). The nymphs were sampled under current velocity ranging from 0.24 to 0.48 m/sec, the average streams width from 60 cm to 1.50 m, with depth from 10 to 35 cm, and pH from 6.8 to 7.2. The highest population density was recorded at the Charchar site (60 individuals/m^2^) and the lowest one was observed at the Bouailef site (2–5 individuals/m^2^).

The mature nymphs and subimagos (together with early-instar nymphs) were observed in May/June and another generation observed in September/October, thus suggesting a bivoltine life cycle. The other Ephemeroptera species sporadically occurring in the same sites were *Caenisluctuosa* (Burmeister, 1839), *Baetischelif* Soldan, Godunko & Thomas, 2005 and *Baetissinespinosus* Soldán & Thomas, 1983.

## Supplementary Material

XML Treatment for Ecdyonurus aurasius
